# Characteristics of a Lipid Hydrogel and Bigel as Matrices for Ascorbic Acid Stabilization

**DOI:** 10.3390/gels9080649

**Published:** 2023-08-11

**Authors:** Noèlia Loza-Rodríguez, Aina Millán-Sánchez, Olga López

**Affiliations:** 1 Department of Chemical and Surfactant Technology, Institute of Advanced Chemistry of Catalonia (IQAC-CSIC), C/Jordi Girona 18-26, 08034 Barcelona, Spain; aina.millan@iqac.csic.es; 2 Bicosome S.L., C/Jordi Girona 18-26, 08034 Barcelona, Spain

**Keywords:** bigel, colloidal lipid hydrogel, ascorbic acid, stability study, rheology, texture analysis, nanostructural analysis

## Abstract

Ascorbic acid (AA) has many health benefits, including immune and cardiovascular deficiency protection, prenatal problems, and skin diseases. Unfortunately, AA is easily oxidized and has limited bioavailability. Thus, the development of formulations that stabilize and enhance the efficacy of AA is a challenge. In this study, 4% AA was encapsulated in two recently developed gels, a hydrogel and a bigel. The hydrogel was formed exclusively with lipids and water, and the bigel was a combination of the hydrogel with an oleogel formed with olive oil and beeswax. The effect of AA in gel microstructures was determined using X-ray scattering, rheology, and texture analysis. Additionally, the capacity of these materials to protect AA from degradation upon temperature and sunlight was studied. Results showed that the incorporation of AA into both materials did not affect their microstructure. Moreover, hydrogel-protected AA showed only 2% degradation after three months at 8 °C, while in aqueous solution, it degraded by 12%. Regarding sunlight, bigel showed a good shielding effect, exhibiting only 2% AA degradation after 22 h of exposure, whereas in aqueous solution, AA degraded by 10%. These results suggest that both proposed gels could be used in biomedical applications and the field of food.

## 1. Introduction

Ascorbic acid (AA), also known as Vitamin C, is not only essential in many metabolic pathways [1,2] but also an important component in endogenous antioxidant defense. It has been demonstrated to be effective as a scavenger of reactive oxygen species, which damage cellular proteins, lipids, and DNA. In the skin, AA plays many important roles, including the formation of the skin barrier and collagen in the dermis and the ability to counteract skin oxidation [3].

A deficiency of AA is associated with many diseases, such as anemia, infections, scurvy, poor wound healing, capillary hemorrhage, muscle degeneration, etc. [4]. It has been reported that AA deficiency can cause the development of some skin diseases, such as atopic dermatitis and porphyria cutanea tarda. In addition, high doses of AA have significantly reduced cancer cell viability, as well as invasiveness, and induced apoptosis in human malignant melanoma [5].

Due to random genetic mutations that occurred during evolution, humans are unable to synthesize AA [6]. Therefore, this molecule must be obtained through the diet. In healthy organisms, a normal diet rich in fruits and vegetables is usually enough to provide the amount of AA necessary to cover physiological requirements. However, in certain situations, dietary and topical supplementation is necessary.

The main challenge in the development of AA products is high instability and reactivity. AA is reversibly oxidized into dehydroascorbic acid (DHA) upon exposure to light, heat, transition metal ions, and pH (alkaline condition). Then, DHA further irreversibly hydrolyzes to form 2,3-diketogulonic acid. During the last decades, a number of research groups have proposed different strategies to decelerate AA oxidation and to study its antioxidant activity, including encapsulation of the molecule in W/O, O/W, and W/O/W emulsions [7,8] and nanoemulsions [9], liposomes [10], niosomes [11,12], acid serums [13], and inorganic nanocapsules [14]. Other approaches are related to synthetizing AA derivatives [15] and formulating with redox partners such as Vitamin E [16]. Thanks to these studies, progress has been made in this matter, although it is necessary to continue working to achieve transport systems capable of keeping AA stable, delivering it accurately to the desired site at the rate and time required.

Our research group has reported several studies regarding lipid materials and skin applications. The latest research is a complete characterization study of a new hydrogel (HG) invention, including a patent reported by K. Talló [17,18,19,20], and a characterization and skin permeation study of a new biocompatible lipid-based bigel (BG) reported by Loza-Rodríguez et al. [21]. The HG is a colloidal lipid gel formed by aggregation of lipid vesicles under dilute conditions and without requiring gelling agents. Their microscopic structure and rheological behavior indicate that the formation and integrity of the HG are related to the attractive–repulsive interactions between the vesicles [17,18,19,20]. In addition, their soft nature and low structural strength make this material a potential candidate for topical applications. On the other hand, the BG is a combination of the aforementioned HG and an oleogel (OG) composed of olive oil (OO) and beeswax (BW). Considering their dual nature, BGs have characteristics of both HGs and OGs and can exhibit a synergistic effect [22,23,24,25,26,27]. These two materials have been shown to interact with the skin and promote the penetration of hydrophilic and lipophilic fluorescence probes into the cutaneous tissue.

In this work, we propose two gel-based materials to protect AA from degradation against temperature and solar radiation, the HG and the BG (as mentioned before in references [17,21]). The different micro- and nanostructural organizations of both HG and BG, along with the fundamental role of water and lipids in their composition, suggest that they can be a suitable matrix to incorporate AA and protect it from degradation. An improvement in this research, compared to other similar BGs, hydrogels, and encapsulation strategies found in the literature [10,13,28,29,30,31,32,33,34], is that our material is composed only of lipids and water. No polymers, resins, acid ascorbic derivatives, or chelating agents (biocompatible or not) are introduced in our formulas. In the BG, the OG is formulated with olive oil (OO) and beeswax (BW), and the HG is nanostructured with rearranged phospholipid vesicles instead of a polymer. Everything confers high biocompatibility to these materials and could enhance skin permeation. Moreover, the BG is a two-phase system with solid-like characteristics, enhancing the stability of the formula compared to emulsions and liposome solutions [23,25]. Therefore, in this study, AA was incorporated into both materials and characterization was carried out through rheology studies, texture analysis (TA), and X-ray scattering. The ability of HG and BG to protect AA was evaluated by comparing the degradation of AA included in these materials or aqueous solution control in different conditions. Our results indicate that both HG and BG exert protection over AA.

## 2. Results and Discussion

### 2.1. Visual Appearance, Perception, and Drug Content

**Figure 1:**
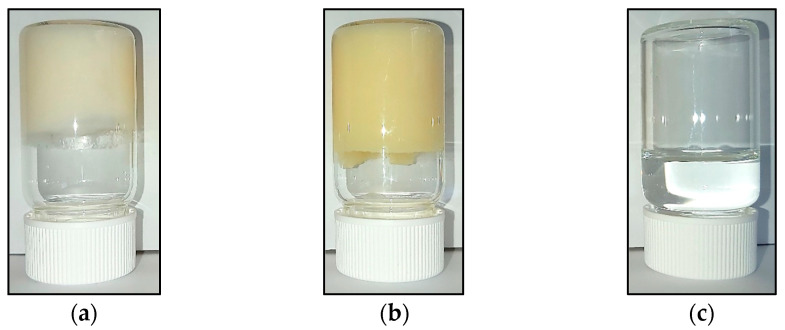
Visual appearance of the materials: (**a**) HG 4% AA, (**b**) BG 4% AA, and (**c**) 4% AA in water.

In Figure 1, the visual appearance of the HG, BG, and aqueous solution containing 4% AA are presented. The gelation was confirmed by the inverted tube test, which showed no gravitational flow in HG (Figure 1a) and BG (Figure 1b) samples. The appearance of HG and BG presented good integrity and homogeneity; HG was a soft creamy white gel, while BG presented higher hardness and a yellow color coming from BW and OO. The ascorbic acid solution was a transparent and colorless liquid (Figure 1c).

### 2.2. Rheological Behaviour

#### 2.2.1. Strain Sweep Assay

The strain sweep measurement was used to determine the Linear Viscoelastic Region (LVR) at a relatively low (1.0 Hz) frequency. Moreover, it was a way to follow the flow behavior of the systems under large deformation.

**Figure 2:**
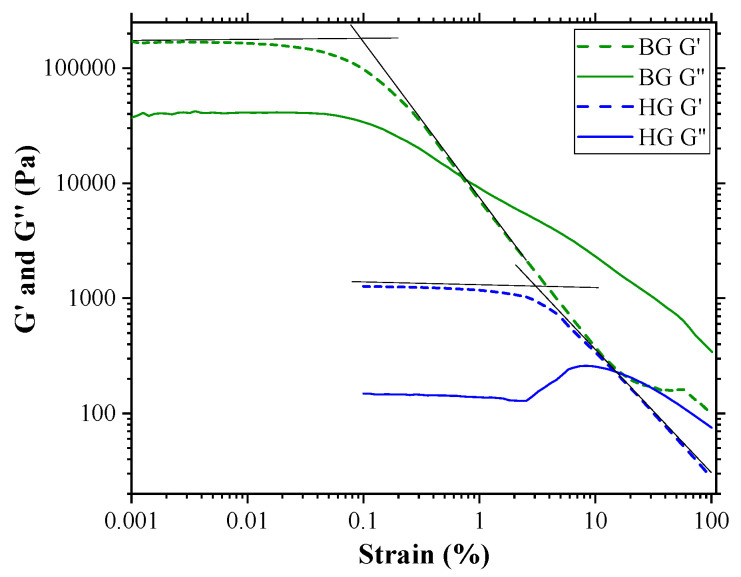
Strain sweep test of HG 4% AA and BG 4% AA samples. Elastic modulus (G′) and viscous modulus (G″) are shown as a function of strain. The critical strain corresponds with the intersection of the two tangents (black lines).

Figure 2 shows the corresponding variation of elastic modulus (G′) and viscous modulus (G″) with increasing oscillation strain. BG and HG exhibit a single plateau region known as LVR in which both moduli remain linear. In this region, G′ values were always larger than G″ values, indicating that the materials were highly structured. Comparing the values of the moduli in this region, we clearly observe that they are higher for the BG than for the HG. This fact indicates a higher resistance of BG to elastic deformation.

The decrease in the G′ moduli indicates the break point of the gel network structure. This point is identified as the critical strain, and it is estimated by the intersection of the two tangents. These values were 3.2% and 0.1% strain for HG and BG, respectively. Considering that the critical strain indicates how resistant a gel is to being broken, our data point to HG showing more resistance to being unstructured than BG, despite BG being more resistant to elastic deformation, as mentioned above.

The behavior of G″ beyond the critical strain was different for the two materials studied. In the case of BG, G″ decreases and crosses the elastic modulus, indicating that viscous behavior is dominant at higher strain. On the contrary, for HG, once the critical strain is overcome, an initial rise of G″ and then a decrease is observed, exhibiting a strain overshoot behavior. This phenomenon could be attributed to the formation of a weak intermediate structure that resists deformation until a certain strain value is reached. Above this value, the intermediate structure is broken, leading to the flow of the material, and G″ becomes higher than G′ [35,36]. The strain overshoot occurs in many systems, including polymer solutions, emulsions, soft glasses, particle suspensions, and hydrogels [37,38]. It is noteworthy that this behavior of G″ in the HG with AA was not observed in previous studies by K. Talló et al., where HG did not contain AA [20]. AA is a molecule that provides ionic charge to the material. Since HG is a material where vesicle aggregation is governed by ionic forces, the addition of AA could modify the aggregation–disaggregation capacity of the vesicles, leading to the peculiar behavior of the viscous module.

#### 2.2.2. Frequency Sweep Assay

**Figure 3:**
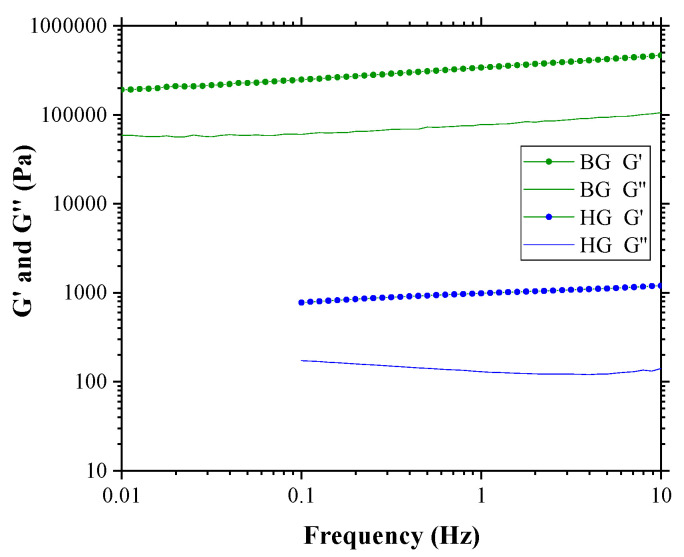
Frequency sweep test of HG 4% AA and BG 4% AA samples at constant strains of 0.5% and 0.005%, respectively.

Figure 3 shows the variation of G′ and G″ with increasing oscillation frequency. The strain was kept constant at the LVR of both materials, which was 0.5% for HG and 0.005% for BG, respectively.

The elastic modulus from both gels is higher than the viscous along the frequency range from 0.01 to 10 Hz for both gels, indicating a typical weak gel behavior [32]. According to Chaux-Gutierrez et al. [39] and Patel et al. [40], gels can be classified into two types: true-gels and weak-gels. In true-gels, G′ and G″ are independent of the frequency. In weak-gels, G′ and G″ are dependent on the frequency and G′ > G″. According to this classification, BG and HG are considered weak-gels as G′ > G″ and G′ and G″ decrease towards smaller frequencies, being dependent on the frequency oscillation [20,32,39,40,41]. Nevertheless, BG has more solid-like characteristics than HG. The BG is harder than the HG due to the OG matrix, making it initially more resistant to deformation. However, once deformed, it becomes easier to break. It must be considered that the BG is composed of an OG matrix with the HG embedded within it [21]. At a microstructural level, the BG exhibits a certain heterogeneity that may facilitate its fracture or separation. On the other hand, the HG is softer but possesses a more uniform microstructure [20,21], which allows it greater resistance to breakage.

It is interesting to note that the values of G′ and G″ obtained in the LVR for these two materials (BG: G′~1.7 × 10^5^ Pa, G″~4.0 × 10^4^ Pa and HG: G′~1.2 × 10^3^ Pa, G″~1.4 × 10^2^ Pa) coincide with different multi-component systems, based on lecithin, fatty acids, alcohols, esters, and natural waxes. For example, according to Shakeel et al. [42], the behavior of BG is very similar to canola oil systems with lecithin/ethylcellulose or lecithin/sitosterol or systems containing sunflower oil and monoglycerides. On the other hand, the rheological behavior of HG is similar to systems containing sunflower oil with lecithin and sucrose esters or wax [42]. It should be noted that BG and HG presented in this work are new. These materials could replace other systems that are already being used in the food industry. Applications in the field of biomedicine could also be considered, given the biocompatible nature of the components.

Comparing the materials proposed in this study, in which AA has been included, with the same materials without AA (recently published results [21]), we can state that the incorporation of this molecule at the concentrations under study does not interfere with its preparation nor its rheological characteristics in a relevant way.

### 2.3. Texture Analysis

Texture analysis (TA) is a technique with applicability in many different fields, such as food [41,43,44], cosmetic, and pharmaceutical industries for product or formula characterization [45,46]. Through a force vs. time graph obtained from a compression test, different textural parameters can be obtained: hardness, consistency, cohesiveness, and index of viscosity.

**Figure 4:**
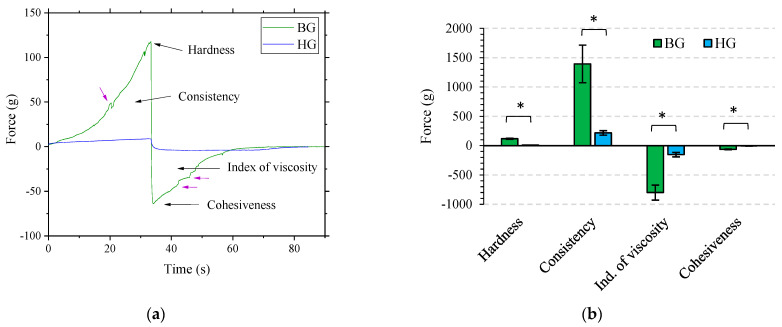
(**a**) Texture analysis plot from the compression test of BG 4% AA and HG 4% AA, with data plotted as force (g) vs. time (s). (**b**) Results of hardness, consistency, index of viscosity, and cohesiveness of both gels. Force values are expressed as mean ± SD of n = 3 gels with triplicate analysis each. Significant differences between materials are represented with an asterisk.

Figure 4a shows the TA compression test profile for BG and HG with 4% AA, and Figure 4b compares BG and HG TA results with the standard deviation and its significant differences for each parameter. Hardness is the necessary force to reach a certain deformation. Consistency is the higher force needed to insert the probe into the sample. Cohesiveness or adhesiveness is a property related to the strength required to overcome the attractive forces between the surface of the gel and the probe during decompression. The index of viscosity is the resistance to removing the probe from the sample [45]. In this compression test, hardness is obtained from the maximum value of the positive curve and consistency from the positive area under the curve (Figure 4a). Cohesiveness is the maximum value of the negative curve, and the index of viscosity is the area under the negative curve (Figure 4a) [45].

Through visual analysis of graphs, the formulation that stands out due to its higher absolute values of hardness, consistency, index of viscosity, and cohesiveness is the BG. The HG is a soft lipid-gel, as rheological study showed, and BG is a mixture of 40% HG and 60% OG with properties clearly given by the presence of 6% of BW (see Table 1) according to Loza-Rodríguez et al. [21]. This previous work reported that BG’s nanostructure was strongly influenced by the BW content in the OG. TA results in Figure 4b show significant differences between HG and BG in all texture properties. These results correlate with rheology analysis, which stated that BG had a higher resistance to elastic deformation and a more solid-like behavior than HG.

The texture profile of the BG also provides useful information. In addition to the fact that the index of viscosity for BG is also higher, the shape of the positive and negative curves became a non-monotonous line and displayed the presence of unsolved shoulders (see purple arrows from Figure 4a). This suggests that the compression and the withdrawal of the probe from the formula consist of several “steps” related to the heterogeneous microstructure of the BG [45]. This material heterogeneity was also found in the rheology strain sweep section (Figure 2) and is in agreement with a previous work in which the BG was prepared in the absence of AA [21]. The BG is highly resistant to deformation; however, once deformed, its heterogeneity may facilitate faster fracture compared to the HG.

### 2.4. Small and Wide-Angle X-ray Scattering (SAXS/WAXS)

**Figure 5:**
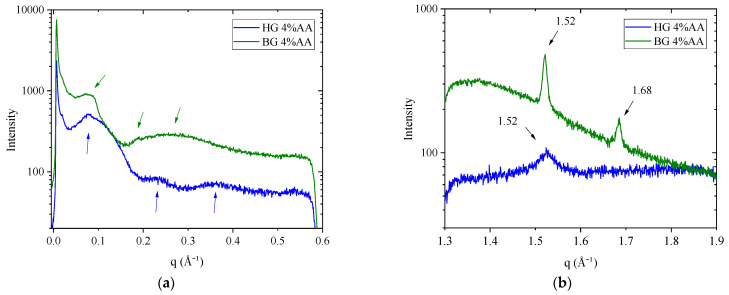
(**a**) Small-angle X-ray scattering (SAXS) and (**b**) Wide-angle X-ray scattering (WAXS) intensity profiles of the HG and BG with 4% AA.

X-ray scattering has been used to study the phase behavior and organization of the HG and BG containing AA. SAXS provides information about the arrangement of the molecules (lipids, AA, and water) and the crystallinity inside the bulk of the HG. For the BG samples, SAXS spectra give information about crystal structure and the way these crystals are organized in the bulk of the material. Otherwise, WAXS spectra report lateral packing organization, providing the distance between neighboring molecules. Figure 5 shows SAXS and WAXS profiles of HG and BG containing 4% AA.

HG sample shows a SAXS profile (Figure 5a) with a main wide band centered approximately at a q value of 0.08 Å^−1^, followed by two additional lobes at 0.23 Å^−1^ and 0.36 Å^−1^. These q values correspond to Bragg’s distances of 8.0 nm, 2.6 nm, and 1.7 nm, respectively. These q and d-spacing values are in line with data reported by Talló et al. [19,20] for the same HG but without the incorporation of AA. The shape of the scattering profile is typical of a scattering form factor corresponding to uncorrelated bilayers. This is expected, given that in the HG, phospholipids are dispersed in high diluted aqueous solution and self-assemble in vesicles [19,20].

SAXS spectra of the BG sample (Figure 5a) presented a single, small, and wide reflection between 0.07 and 0.09 Å^−1^, followed by a deep valley, a weak peak at ≈0.19 Å^−1^, and finally a broad band at ≈0.26 Å^−1^. These q values correspond to Bragg’s distances of 8.6 nm, 3.3 nm, and 2.45 nm, respectively, and are in agreement with data reported previously for BG without AA [21], indicating no alterations in the lipid arrangement due to the effect of the AA. According to A.J. Martins et al. [47], two reflections with a 1:2 relation, with the first q value around 0.09 Å^−1^, appeared in SAXS spectra of mixtures of TAGs and beeswax oleogels, indicating a lamellar organization.

WAXS spectra report lateral packing organization, providing the distance between neighboring molecules. WAXS plot for the HG (Figure 5b) showed a single broad band centered at q = 1.52 Å^−1^, corresponding to a d-spacing of 4.12 Å. This reflection, around 4.1–4.2 Å, is associated with the presence of a lamellar gel phase organized in a two-dimensional hexagonal lateral packing [19,48].

The WAXS plot for BG (Figure 5b) showed two peaks in q ≈ 1.52 and q ≈ 1.68, corresponding to d-spacings of 4.14 Å and 3.74 Å, respectively. These sharp peaks match with β′ polymorphic wax crystal structures, which have an orthorhombic subcell structure. This reflection in WAXS profiles was described in an equivalent BG without AA [21].

Our SAXS and WAXS experiments indicate that the incorporation of AA into both HG and BG leads to a characteristic structural organization. Although some studies have reported that gels produced by water dispersions of AA may undergo alterations involving changes in the SAXS pattern and rheology [49], compared with our previous studies, our results indicate that both BG and HG retain their characteristics even in the presence of AA.

### 2.5. Thermal and Light Stability of Ascorbic Acid

The capability of BG and HG to prevent AA degradation under storage at different temperatures and under solar radiation exposure has been studied.

Throughout the entire results section, AA stability is represented as a % of AA degradation, as the values have been normalized with the AA concentration at the initial point of the experiment. Additionally, two appendices, Appendix A for temperature stability and Appendix B for photostability study, are provided at the end of the manuscript. They provide information about the corresponding amount of AA as a function of time and also *p*-values to determine the significant differences in %AA degradation between materials. Table A1 represents the content of AA over time at three different temperatures for all three systems. Table A2 represents the *p*-values for the temperature stability study. Table A3 shows the content of AA after different times of irradiation, and Table A4 shows the *p*-values for photostability studies.

#### 2.5.1. Temperature Stability

**Figure 6:**
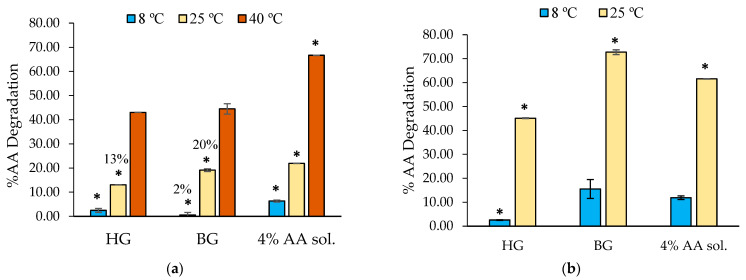
Degradation of AA incorporated in HG, BG, and a water solution stored for (**a**) 1 month at 8 °C, 25 °C, and 40 °C and (**b**) 3 months at 8 °C and 25 °C (8B). Significant differences between materials for each temperature are marked with asterisks, and they were considered with *p*-values ≤ 0.05.

The degradation of AA incorporated in the HG, the BG, and a control solution of water at 4% AA stored at three different temperatures was evaluated. Figure 6 shows the degradation % of AA after 1 month at 8 °C, 25 °C, and 40 °C (Figure 6a) and 3 months at 8 °C and 25 °C (Figure 6b). Values after 3 months at 40 °C are not shown due to structural destabilization of both gels stored at these conditions. The AA degradation differences between materials were evaluated for each temperature with one-way ANOVA.

After 1 month and regardless of the temperature, AA was better preserved when it was incorporated into HG and BG than when it remained in the aqueous solution (Figure 6a). At 8 °C (1 month), the system that best protects AA, with only 2% degradation, is the BG. However, the HG showed a better stabilization at 25 °C compared to the BG, with AA being 13% degraded in HG and 20% in BG. After 1 month at 40 °C, the degradation of AA in any of the samples was high. Nevertheless, we can observe that its incorporation in HG or BG helps to preserve this molecule from degradation, compared to the results of the aqueous solution (~44% degradation in HG or BG and 66% in water).

After 3 months (Figure 6b), results showed that the HG protects AA at 8 °C and 25 °C better than BG and water. Curiously, our results indicated higher AA degradation at 25 °C when included in BG compared with HG and the control water solution. We believe that these results can be attributed to the distinctive microstructure of the material.

The HG and BG microstructures were previously described by Talló et al. [17] and Loza-Rodríguez et al. [21] in previous research. Talló et al. described the HG as a colloidal HG made only of lipids and water with a supramolecular aggregation of lipid multilamellar vesicles coexisting with lipid lamellae. Loza-Rodríguez et al. described the BG as a distribution of little HG domains (of this previously mentioned HG) inside a continuous OG matrix. In this study, AA is incorporated in the aqueous part of the HG, and in the BG, the AA is inside the small domains of HG that are included in the matrix of OG.

In any case, both in the HG and in the BG, the AA is in contact with water and with the lipid bilayers. Considering that lipid bilayers have a slightly positive ionic character due to the presence of DOTAP, an interaction of the ascorbate ion with lipids, in addition to water, would be expected. This fact would take place in the HG and the BG but not in the AA system in aqueous solution. According to Golonka et al. [34], the photostability of AA and its derivatives in a hydrophilic environment is related to the substance–polymer interaction. Perhaps, in our formulas, this ionic interaction with the lipid bilayer and ascorbate ion could be related to the ability to stabilize AA that we observe.

However, regarding the BG results, according to Uluata et al. and Noon et al., lipids tend to undergo oxidation over time [50,51], and different molecules have been studied to combat lipid oxidation. AA could work as an antioxidant in the systems [50,51], and it might be degraded at the expense of the lipids in our systems. Given that BG has a higher concentration of lipids due to OG content in the formula, a higher degradation of AA would be observed.

#### 2.5.2. Photostability Study

In this section, the stability of AA encapsulated in the HG, the BG, and a control solution of water at 4% AA was conducted. These tests were carried out at two different exposure times.

**Figure 7:**
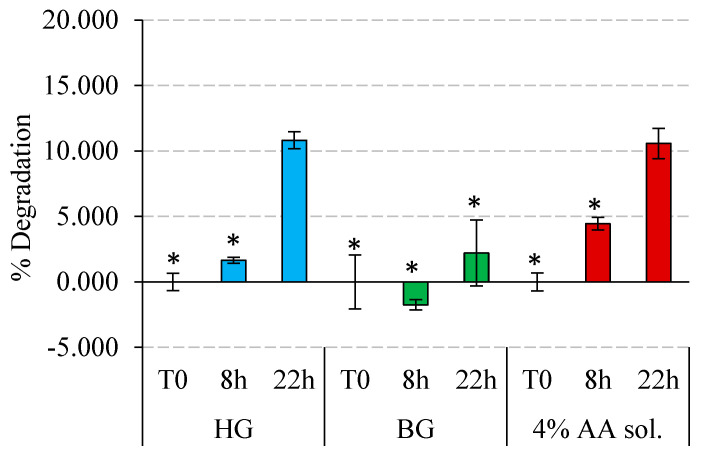
Stability of HG 4% AA, BG 4% AA, and AA solution 4% under light radiation. Degradation of AA in % is represented at different exposure times for each material. Significant differences between materials for each exposure time are marked with asterisks, and they were considered with *p*-values ≤ 0.05.

Figure 7 illustrates the results of AA degradation after 8 and 22 h of light exposure. In general, for all the systems, a longer exposure time induces a greater degradation. Remarkably, BG revealed a high protective effect, avoiding AA degradation after 8 h of exposure and showing only 2% degradation after 22 h of exposure. Under the same conditions but included in the HG and solubilized in water, AA experienced a much higher degradation, around 10% after 22 h. In any case, after 8 and 22 h under light radiation, the HG protected better than the aqueous solution.

The behavior of HG and BG as AA protectors could be related to the ability of these materials to absorb radiation at wavelengths in the range of 310 nm to 800 nm. In the HG, AA is likely located in the aqueous part, and the lipids forming the HG structure (5% total lipid content) would have a reduced ability to absorb light. Consequently, this material has a limited potential to protect the AA from radiation due to direct exposure. Conversely, in the BG, the presence of the OG matrix (formed by olive oil and beeswax) would act as a barrier, absorbing part of the light radiation and preventing it from reaching the HG domains within where the AA is located. The absorption reported for the beeswax is in the range of 270–400 nm [52], and for the olive oil, between 350 and 600 nm [53]. This fact would have an important role in the ability of the BG to absorb light and, consequently, protect the AA. In addition, the compact structure of the BG, as observed in texture analysis from Figure 4a,b, may further contribute to this shielding effect against light radiation.

Our results indicate that both HG and BG are good candidates to protect AA from thermal degradation and from that induced by exposure to light. However, the response of these two materials against degradation-inducing phenomena was different. HG preserves better against temperature, while BG is better against exposure to light.

Given that in the two materials, the AA is located in the HG, intuitively, a similar behavior with respect to temperature would be expected. The superior protection of HG could be related to the antioxidant capacity of AA itself, which would be more required in BG due to its higher lipid composition, as mentioned above. Possibly, the addition of other antioxidant molecules in the BG would enhance the performance of this material. Future studies should address these challenges.

## 3. Conclusions

In this work, we proposed two gel-based materials, an HG and a BG, to protect AA from degradation against temperature and solar radiation. The characterization results showed that the incorporation of 4% AA did not interfere with the rheological characteristics nor the nanostructure of the materials in a relevant way. The ability of both HG and BG to protect AA indicates that they both contribute to the stabilization of AA against degradation. In conclusion, the two proposed materials are capable of reducing the degradation of AA in different situations and the structure, composition, and rheological behavior of the two gels could be compatible with their use both in biomedical applications and in the field of food.

## 4. Materials and Methods

### 4.1. Materials

Hydrogenated soy phosphatidylcholine (HSPC; Phospholipon^®^ 90H) and 1,2-dioleoyl-3-trimethylammonium-propane (DOTAP) were supplied from Lipoid GmbH (Ludwigshafen, Germany). Extra Virgin Olive Oil (OO) was obtained from Aceites del Sur—Coosur, S.A. (Vilches, Jaén, Spain), and beeswax (BW) was purchased from EsentialArôms Dietéticos Intersa, S.A. (Alcarràs, Lleida, Spain). Chloroform was supplied from Carlo Erba Reagents S.A.S. (Val de Reuil, Paris, France), α-Tocopherol acetate and ascorbic acid from Guinama S.L.U (La pobla de Vallbona, Valencia, Spain), and purified water was obtained by an ultrapure water system, Milli-Q plus 185 (Millipore, Bedford, MA, USA). Methanol for liquid chromatography and orthophosphoric acid were supplied by Sigma Aldrich (Merck KGaA, Darmstadt, Germany).

### 4.2. Gel Preparation

#### 4.2.1. Hydrogel

HG was prepared following the film hydration method, according to the literature [18,20]. In summary, HSPC and DOTAP were solubilized with chloroform in a round bottom flask and slowly evaporated with a rota-evaporation system to obtain a lipid film. Afterwards, the film was hydrated with an aqueous solution of ascorbic acid using an ultrasound bath at 25 °C. Then, a temperature cycle was performed in a closed vial: first, it was frozen at −20 °C for 3–4 h, then heated at 70 °C for 10 min, and finally, the solution was left to cool at 5 °C in the fridge to obtain gelation. Gelation was confirmed by the absence of gravitational flow when the test tubes containing the HGs were inverted, the so-called “inversion test”. Two HGs were prepared, one with 10% AA and the other with 4% AA. The most concentrated HG was used to form the BG (see below). The concentration of each component is detailed in Table 1.

#### 4.2.2. Bigel

First, OG and HG were prepared separately. OG was prepared by weighing together OO, BW, and Tocopherol. Then, they were heated at 70 °C to obtain a fluid material. HG with 10% AA was also heated at 70 °C. To obtain the final BG 4% AA, the OG was mixed with the HG 10% AA at 70 °C with the proportions 40% HG and 60% OG, stirring for 10 min at 1100 rpm with a magnetic bar. Finally, the mixture was left to cool to 25 °C. Gelation was confirmed by the inversion test, and the absence of phase separation indicated the correct formation of the BG.

### 4.3. Gel Characterization

#### 4.3.1. Rheology

Oscillatory rheology tests of the HG and BG were performed with an AR-G2 controlled stress rheometer (TA Instruments, New Castle, DE, USA) equipped with a Peltier temperature control system. Parallel plate geometry of 20 mm diameter and a gap of 2000 µm and 3000 µm were used for HG and BG, respectively. Oscillation amplitude measurements were used to determine the LVR of both materials. Then, frequency measurements were performed within the LVR to ensure that the material response in terms of elastic modulus (G′) and viscous modulus (G″) was independent of the strain magnitude. Data were analyzed using TRIOS software 5.2 (TA Instruments, New Castle, DE, USA), and final data values were represented with Origin.

G′ is related to the stored energy, while G″ represents the dissipated energy. Critical strain was defined as the intersection of the tangents from the baseline of the linear region and initial slope from the non-linear region. During the strain sweep, the frequency was kept constant at 1 Hz, and during the frequency sweep, the strain was kept constant at 0.5% and 0.005% for HG and BG, respectively. Samples were evaluated in triplicate at 25 °C.

#### 4.3.2. Texture Analysis

The texture profile analysis on the materials was conducted using a Texture Analyzer MT-LQ (TA) from Stable Microsystems. It was equipped with a 5 kg interchangeable load cell for TA-I-Di Texture analyses. The method consisted of inserting the analytical probe into the sample, measuring the penetration depth of a cylindrical probe P0.05 R at a constant force, leading to a predefined period of recovery, resulting in a force (g) versus time (sec) graph. Different parameters were evaluated: the hardness of the material, firmness, cohesiveness, and the index of viscosity. The test was set with a 10 mm compression displacement with a 0.3 mm/s test speed and a 3 g trigger force. The gels were placed centrally under the probe, adjusted until just above the sample. All of them were untouched and recently prepared, and the vial was fixed to avoid displacement provoked by the stickiness of the material. Samples were evaluated in triplicate at 25 °C.

#### 4.3.3. Small- and Wide-Angle X-ray Scattering (SAXS/WAXS)

The SAXS and WAXS measurements were performed using the SAXS/WAXS S3-MICRO (Hecus X-ray Systems GmbH, Graz, Austria) in combination with a GENIX-Fox 3D X-ray source (Xenocs, Grenoble, France) with a wavelength of 1.542 Å (CuKα line). The linear detector was a PSD 50 M (Hecus, Graz, Austria), and the temperature was controlled using the Peltier TCCS-3 (Hecus, Graz, Austria) with a precision of ±0.1 °C.

Measurements of SAXS and WAXS were performed with sample-to-detector distances of 268 mm and 282 mm, respectively. Samples were sandwiched between two aluminum cells with Mylar windows that sealed the sample to allow the beam to pass directly through it. Measurements were carried out at 25 °C under vacuum.

The intensity of scattering I (expressed in arbitrary units) was determined by measuring the scattering vector q that resulted from the sample being exposed to a photon of wavelength *λ* and scattered at an angle of 2 *θ*, following the Equation (1) [54]:

(1) $$q=\frac{4\pi \;sin\theta }{\lambda }$$

Finally, the Bragg’s distance (Equation (2)) was calculated with:

(2) $$D_{Bragg=}\frac{n2\pi }{q}$$

In a lamellar organization, the various peaks are located at equidistant positions, and *Qn* in Equation (3) represents the position of the nth-order reflection [54].

(3) $$Qn=\frac{2\pi n}{d}$$

ORIGIN Pro 2019 software was used for data analysis and acquisition of *q* values.

#### 4.3.4. Ascorbic Acid Stability Studies

##### Thermal Stability

The ability of HG and BG to protect AA from degradation against temperature was evaluated. For this, samples of these materials containing 4% AA were exposed to 8 °C, 25 °C, and 40 °C, and the results were compared with the behavior of AA in aqueous solution under the same conditions (Table 2). The tests were carried out in chambers with temperature control, 65% humidity, and without light exposure.

AA was extracted from the samples and analyzed by High-Performance Liquid Chromatography (HPLC). The following time schedule was pursued to analyze the samples.

Sample treatment varied depending on the material. For the HG sample, 0.05 g was weighed in a 10 mL volumetric flask and dissolved with MeOH:H_2_O (2:8).

For the BG sample, an additional extraction had to be made. First of all, 0.05 g was weighed and dissolved with CHCl_3_:MeOH (8:2). Afterwards, it was placed in a 15 mL centrifuge tube, and three extractions with 4 mL, 2 mL, and 1 mL of water were performed. Centrifuge conditions were 4000 rpm and 2 min, using an NF 200 Bench Top centrifuge (Nüve, Ankara, Turkey). Extracted water was placed in a 10 mL volumetric flask, which was finally levelled with MeOH. All samples were filtered using Nylon Syringe filters with 0.22 µm pore size before being transferred to the autosampler.

Duplicate samples were analyzed, and each vial was injected twice. In the results section, data are represented by % of AA degradation.

The chromatographic analyses were performed using an HPLC HP1260 Infinity II (Agilent Technologies, Santa Clara, CA, USA). The apparatus consisted of a binary pump (G7112B), autosampler (G7129A), and DAD detector (G7117C). The system was operated using the software OpenLab CDS 2.1 (Agilent Technologies, Santa Clara, CA, USA).

Mobile phases were 20% Methanol and 80% phosphate buffer (MilliQ water with 0.1% H_3_P0_4_). The column was a ZORBAZ Eclipse Plus C18. The analysis was performed at 25 °C. A total of 5 µL of the sample was injected, and the signal was detected at ʎ = 244 nm.

To determine the final concentration of our materials, a calibration curve was constructed using a standard solution of AA. Two stock solutions were prepared, and subsequent dilutions were made accordingly to achieve the following concentrations: 25, 50, 100, 150, 200, 250, and 500 ppm. Two replicates of each vial were injected. The analytical methodology was previously validated in terms of the calibration curve, limit of detection, limit of quantitation, and precision.

##### Light Radiation Stability

In total, 0.4 g of BG, HG, and water solution containing 4% AA were introduced into 5 mL transparent closed vials and exposed to a light source simulating solar radiation (Suntest CPS+, Atlas, Mount Prospect, IL, USA). The samples were subjected to 8 h and 22 h of 500 Wm-2 of radiation in the range of 310 nm to 800 nm, which corresponds to UVA from 310–400, VIS from 400–760 nm, and a small region of IRA from 760–800 nm. This radiation intensity is equivalent to the radiation exposure of 12 h and 36 h in June in Catalonia [55]. The maximum temperature reached in the simulator was 35 °C [56]. After the exposure, the AA was extracted and analyzed by HPLC.

#### 4.3.5. Statistical Analysis

All quantitative data were analyzed at least in triplicate. Values are expressed as the average ± standard error. Standard deviations were calculated for all mean values. Analysis of one-way ANOVA was applied for group comparisons in texture analyzer and stability tests. Significant differences were considered with *p*-values ≤ 0.05.

## Figures and Tables

**Table 1:** Composition of HGs and BG.

Material	Hydrogel 10% AA	Hydrogel 4% AA	Bigel 4% AA
	% *w*/*w*	% *w*/*w*	% *w*/*w*
Water	85	91	34
HSPC	4.8	4.8	1.9
DOTAP	0.2	0.2	0.1
Ascorbic Acid	10	4	4
OO	-	-	53.4
BW	-	-	6
α-Tocopherol	-	-	0.6

**Table 2:** Schedule for the evaluation of AA degradation due to the effect of temperature in the different samples (T_0_: initial time; M: month/s).

8 °C	25 °C	40 °C
T_0_	T_0_	T_0_
1 M	1 M	1 M
3 M	3 M	-

## Data Availability

The data presented in this study are available on request from the corresponding author. The data are not publicly available due to privacy restrictions.

## Appendix A. Temperature Stability

**Table A1:** Concentration of % AA in HG, BG, and AA aqueous solution after different times at three different temperatures. Values are represented as means ± SD.

Material	T_0_			1 Month			3 Months	
	8 °C	25 °C	40 °C	8 °C	25 °C	40 °C	8 °C	25 °C
HG	4.13 ± 0.03	4.13 ± 0.03	4.07 ± 0.01	4.02 ± 0.03	3.59 ± 0.00	2.32 ± 0.00	4.02 ± 0.00	2.26 ± 0.01
BG	3.85 ± 0.08	3.85 ± 0.08	3.95 ± 0.08	3.83 ± 0.04	3.12 ± 0.02	2.19 ± 0.09	3.25 ± 0.05	1.05 ± 0.04
4% AA sol.	4.07 ± 0.03	4.07 ± 0.03	4.07 ± 0.03	3.81 ± 0.02	3.17 ± 0.00	1.36 ± 0.00	3.58 ± 0.03	1.56 ± 0.00

**Table A2:** Summary of *p*-values from one-way ANOVA analysis to determine the significant differences of %AA degradation between materials in the temperature stability test (see Section 2.5.1). They were obtained by comparing the normalized results coming from Table A1.

Temperature	Time	Material Comparison	*p*-Value	Significant Differences
8 °C	1 month	HG—BG	0.03	Yes
		HG—4% water sol.	1 × 10^−4^	Yes
	3 months	HG—BG	6 × 10^−4^	Yes
		BG—4% water sol.	0.12	No
25 °C	1 month	HG—BG	7.5 × 10^−7^	Yes
		BG—4% water sol.	6 × 10^−5^	Yes
	3 months	HG—BG	2.4 × 10^−9^	Yes
		BG—4% water sol.	5 × 10^−7^	Yes
40 °C	1 month	HG—BG	0.22	No
		BG—4% water sol.	9 × 10^−7^	Yes

## Appendix B. Photostability Study

**Table A3:** Concentration of AA in HG, BG, and AA aqueous solution after different times at three different temperatures. Values are represented as means ± SD.

Material	T_0_	8 h	22 h
HG	4.12 ± 0.03	4.05 ± 0.01	3.67 ± 0.03
BG	3.85 ± 0.08	3.92 ± 0.02	3.76 ± 0.10
4% AA sol.	4.07 ± 0.03	3.88 ± 0.02	3.64 ± 0.05

**Table A4:** Summary of *p*-values from one-way ANOVA analysis to determine the significant differences of %AA degradation between materials in the photostability study (see Section 2.5.2). They were obtained by comparing the normalized results coming from Table A3.

Time	Material Comparison	*p*-Value	Significant Differences
8 h	HG—BG	6.2 × 10^−6^	Yes
	HG—4% water sol.	4.5 × 10^−5^	Yes
22 h	HG—BG	5.7 × 10^−4^	Yes
	HG—4% water sol.	0.72	No
